# Caveolin-1 at the Crossroads of Diabetes and Alzheimer’s Disease: New Mechanisms, Biomarkers, and Therapeutic Opportunities

**DOI:** 10.3390/biomedicines14081709

**Published:** 2026-07-30

**Authors:** Andrei Surguchov

**Affiliations:** Department of Neurology, University of Kansas Medical Center, 3901 Rainbow Boulevard, Kansas City, KS 66160, USA; asurguchov@kumc.edu

**Keywords:** caveolin-1, Alzheimer’s disease, diabetes mellitus, insulin resistance, mitochondria, endoplasmic reticulum, neurovascular unit, amyloid beta, tau, neurodegeneration

## Abstract

Type 2 diabetes mellitus (T2D) is increasingly recognized as a major risk factor for Alzheimer’s disease (AD), supporting the concept that chronic metabolic dysfunction contributes to neurodegeneration. Recent advances have identified caveolin-1 (CAV-1), the principal structural protein of caveolae, as an important regulator of insulin signaling, lipid metabolism, mitochondrial homeostasis, neurovascular integrity, and amyloid precursor protein processing. Since our previous review published in 2020, substantial evidence has demonstrated that altered CAV-1 expression and function are associated with AD-related pathology under diabetic conditions through multiple mechanisms, including endothelial dysfunction, impaired brain insulin signaling, disruption of mitochondria–endoplasmic reticulum contact sites (MERCSs), neuroinflammation, mitochondrial dysfunction, and defective autophagy. Experimental studies further show that restoring neuronal or endothelial CAV-1 expression improves insulin signaling, preserves synaptic function, attenuates amyloid pathology, and ameliorates cognitive decline in preclinical models. This review summarizes recent advances in understanding of the CAV-1-dependent mechanisms linking T2D and AD and discusses the emerging potential of CAV-1 as a biomarker and therapeutic target for diabetes-associated neurodegeneration. Collectively, current evidence identifies CAV-1 as a central molecular hub integrating metabolic, vascular, and neurodegenerative pathways and supports its further investigation as a promising therapeutic target.

## 1. Introduction

Alzheimer’s disease (AD) and type 2 diabetes mellitus (T2D) are among the most prevalent age-associated disorders worldwide and represent major public health challenges due to their increasing incidence in aging populations [1,2,3]. AD is the leading cause of dementia and is characterized by progressive cognitive decline accompanied by extracellular amyloid-β (Aβ) deposition, intracellular neurofibrillary tangles composed of hyperphosphorylated tau, synaptic dysfunction, and neurodegeneration. T2D, in turn, is a complex metabolic disease characterized by insulin resistance, hyperglycemia, and chronic low-grade inflammation. Accumulating epidemiological and experimental evidence indicates that these two disorders are closely interconnected, leading to the concept of AD as “type 3 diabetes” or a brain-specific insulin-resistant state [4,5,6].

Epidemiological studies consistently demonstrate that T2D increases the risk of cognitive impairment and dementia by approximately 1.5–2-fold [7,8]. Moreover, individuals with diabetes frequently exhibit accelerated cognitive decline, reduced hippocampal volume, and increased cerebral vascular pathology [9,10]. Recent longitudinal studies suggest that diabetes is associated with accelerated progression of components of the amyloid–tau–neurodegeneration (ATN) biomarker cascade during the prodromal stages of AD, supporting the concept that metabolic dysfunction contributes actively to neurodegeneration rather than functioning merely as a comorbidity [11]. Multiple molecular mechanisms have been proposed to explain this association, including impaired insulin signaling, chronic neuroinflammation, oxidative stress, mitochondrial dysfunction, vascular injury, lipid dysregulation, and defective autophagy [11,12,13]. Insulin resistance in the brain disrupts neuronal survival pathways and synaptic plasticity while promoting Aβ accumulation and tau hyperphosphorylation. Hyperglycemia and advanced glycation end products (AGEs) further exacerbate oxidative damage and inflammatory responses, leading to neuronal dysfunction and blood–brain barrier impairment [11,12,13]. Cerebrovascular abnormalities frequently observed in diabetes also contribute to reduced cerebral perfusion and impaired clearance of neurotoxic proteins.

Increasing evidence positions caveolin-1 (CAV-1) as an important integrator of these pathogenic pathways. CAV-1 is a 21–24 kDa scaffolding protein and the principal structural component of caveolae—specialized cholesterol-rich invaginations of the plasma membrane involved in signal transduction, endocytosis, lipid transport, and mechanosensing [14]. Through its scaffolding domain (amino acids 82–101), CAV-1 interacts with numerous membrane receptors and intracellular signaling proteins, including the insulin receptor, endothelial nitric oxide synthase (eNOS), epidermal growth factor receptor (EGFR), platelet-derived growth factor receptor (PDGFR), vascular endothelial growth factor receptor-2 (VEGFR2), transforming growth factor-β receptors (TGF-βRI/II), Src-family kinases, heterotrimeric G proteins, and components of the PI3K/Akt and MAPK signaling pathways. These interactions regulate receptor localization, kinase activity, nitric oxide production, and downstream signal transduction [15]. CAV-1 is widely expressed in endothelial cells, adipocytes, fibroblasts, astrocytes, and subsets of neurons, placing it at key sites of metabolic and neurovascular regulation [16].

Since our previous review published in 2020 [10], several important advances have substantially expanded our understanding of CAV-1 at the interface between metabolic disease and neurodegeneration. These include the following: (i) growing evidence that diabetes accelerates the amyloid–tau–neurodegeneration (ATN) biomarker cascade in humans [17]; (ii) recognition of endothelial CAV-1 as a regulator of blood–brain barrier (BBB) integrity and cerebral insulin transport [18]; (iii) identification of mitochondria–endoplasmic reticulum contact sites (MERCSs) as critical platforms linking CAV-1 to mitochondrial homeostasis and autophagy [17]; (iv) increasing interest in extracellular vesicle-associated CAV-1 as a potential indicator of neurovascular dysfunction [18]; and (v) preclinical studies demonstrating that restoration of neuronal CAV-1 improves synaptic function and cognitive performance in experimental models [19]. Together, these findings warrant a reappraisal of CAV-1 not only as a structural membrane protein but also as a dynamic regulator integrating metabolic, vascular, and neurodegenerative pathways [20,21,22,23,24,25].

Dysregulation of CAV-1 has been implicated in both diabetes and neurodegenerative diseases. In peripheral tissues, altered CAV-1 expression contributes to adipose tissue dysfunction, insulin resistance, extracellular matrix remodeling, vascular complications, and chronic inflammation. CAV-1 is essential for caveolae formation and maintenance in adipocytes, where it regulates lipid trafficking, insulin receptor signaling, glucose uptake, and membrane homeostasis [23,25]. In the central nervous system, CAV-1 modulates synaptic plasticity, neuroinflammation, mitochondrial function, blood–brain barrier integrity, and amyloid precursor protein (APP) processing [20,22].

Although many experimental studies report reduced neuronal or endothelial CAV-1 expression during aging and in models of diabetes and AD, these findings are not entirely consistent [23,24,25,26,27,28,29,30]. Alterations in CAV-1 expression appear to depend on cell type, tissue, disease stage, brain region, and experimental model. For example, increased CAV-1 expression has been reported in reactive astrocytes and in selected regions of postmortem AD brains, possibly reflecting compensatory remodeling or reactive gliosis, whereas neuronal CAV-1 expression generally declines during neurodegeneration [26]. Similarly, studies in obesity and diabetes have shown that CAV-1 expression may either decrease or increase depending on adipose tissue depot, disease stage, and experimental conditions [21,31]. These observations indicate that CAV-1 is not uniformly up- or down-regulated but rather functions as a dynamically regulated signaling protein whose biological effects are highly context dependent.

Beyond its mechanistic relevance, CAV-1 has attracted considerable interest as a candidate biomarker of disease progression and as a potential therapeutic target in AD, although clinical validation remains limited [18,27,28,29]. Understanding how CAV-1 regulates the crosstalk between T2D and AD may provide new insights into disease pathogenesis and facilitate the development of innovative diagnostic and therapeutic strategies aimed at preventing or slowing neurodegeneration in metabolically vulnerable individuals.

In this review, we summarize recent advances in understanding the role of CAV-1 at the intersection of T2D and AD, with particular emphasis on molecular mechanisms identified since our 2020 review, its emerging biomarker potential, and therapeutic strategies targeting CAV-1-dependent pathways. We propose that CAV-1 represents a central molecular hub linking metabolic dysfunction, vascular injury, and neurodegeneration, thereby providing a unifying framework for understanding diabetes-associated cognitive decline.

## 2. Caveolin-1: Structure and Biological Functions

**Figure 1:**
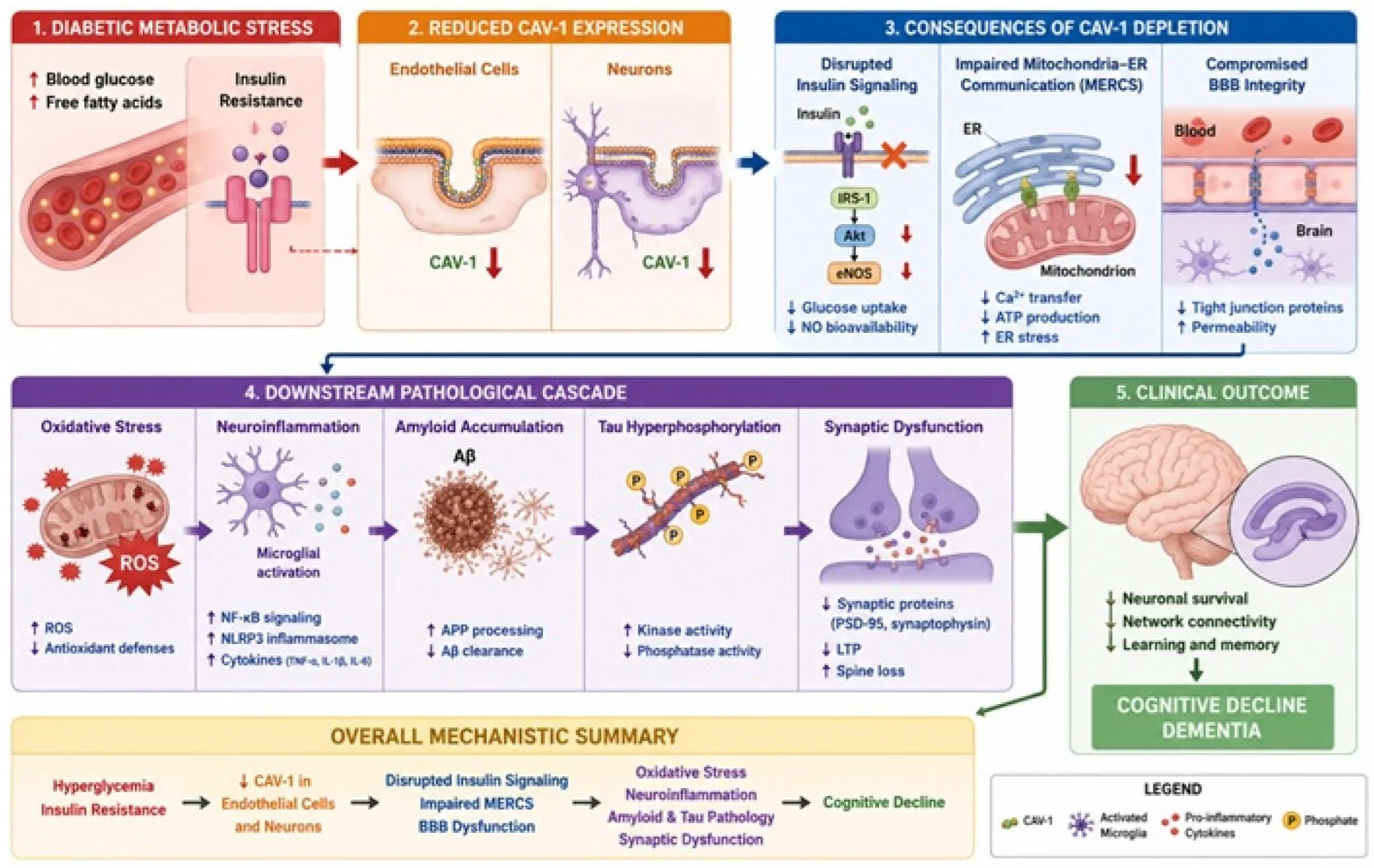
Proposed model illustrating the potential role of CAV-1 dysfunction in linking T2D to A. Hyperglycemia and insulin resistance are proposed to reduce CAV-1 expression and/or function in endothelial cells and neurons. Impaired CAV-1 signaling disrupts insulin signaling, mitochondria–endoplasmic reticulum contact sites (MERCSs), and BBB integrity, thereby promoting oxidative stress, neuroinflammation, amyloid-β (Aβ) accumulation, tau hyperphosphorylation, mitochondrial dysfunction, and synaptic impairment. Collectively, these alterations contribute to neuronal loss, impaired neural network connectivity, cognitive decline, and the progression of AD.

Caveolin-1 (CAV-1) is a multifunctional scaffolding protein that regulates diverse cellular processes through interactions with membrane receptors and intracellular signaling molecules (Figure 1). Through its scaffolding domain, CAV-1 coordinates insulin receptor signaling, cholesterol trafficking, caveolae-mediated endocytosis, mitochondrial homeostasis, inflammatory signaling, autophagy, and synaptic plasticity [21]. The broad expression of CAV-1 in endothelial cells, adipocytes, astrocytes, and subsets of neurons enables it to integrate systemic metabolic regulation with neurovascular and neuronal function [20].

## 3. Evidence Linking CAV-1 to Type 2 Diabetes and Alzheimer’s Disease

Initial experimental evidence supporting a mechanistic role for CAV-1 at the interface between diabetes and AD was provided by Bonds et al., who demonstrated that CAV-1 depletion in diabetic *db*/*db* mice was associated with AD-like pathological changes, including enhanced amyloid precursor protein (APP) processing, an increased Aβ42/Aβ40 ratio, tau hyperphosphorylation, and cognitive impairment [26]. The restoration of CAV-1 expression partially reversed these pathological alterations, suggesting that CAV-1 contributes to maintaining neuronal integrity under diabetic conditions. Subsequent experimental studies have largely corroborated these findings, further supporting the concept that CAV-1 is an important molecular mediator linking metabolic dysfunction to neurodegeneration [23,27].

More recently, longitudinal clinical studies have shown that diabetes is associated with accelerated progression of multiple components of the amyloid–tau–neurodegeneration (ATN) biomarker cascade, reinforcing the concept that chronic metabolic dysfunction contributes to AD pathogenesis rather than merely coexisting with the disease [28]. Although these clinical studies do not directly establish a role for CAV-1, they provide an important pathophysiological framework supporting investigation of CAV-1-dependent mechanisms linking T2D and AD.

## 4. Endothelial CAV-1 and Neurovascular Dysfunction

One of the most important advances since our previous review has been the recognition of endothelial CAV-1 as a critical regulator of neurovascular homeostasis. Brain endothelial cells maintain BBB integrity and regulate the transport of insulin and other circulating molecules into the central nervous system [6,29].

Experimental studies demonstrate that endothelial CAV-1 deficiency impairs cerebrovascular function, disrupts BBB integrity, reduces cerebral insulin transport, and promotes amyloidogenic processing in diabetic models [30]. These findings support the emerging concept that endothelial dysfunction contributes to the early stages of AD pathogenesis. Although direct evidence linking endothelial CAV-1 dysfunction to diabetes-associated AD in humans remains limited, current experimental data place CAV-1 within the neurovascular framework of AD, in which vascular dysfunction is increasingly recognized as an early contributor to neurodegeneration rather than merely a secondary consequence of neuronal injury [31].

## 5. CAV-1 and Mitochondria–Endoplasmic Reticulum Contact Sites (MERCSs)

Mitochondria–endoplasmic reticulum contact sites (MERCSs) have emerged as dynamic signaling platforms that regulate calcium homeostasis, lipid exchange, mitochondrial dynamics, autophagy, mitochondrial quality control, and apoptosis [32]. Increasing evidence indicates that CAV-1 localizes to mitochondria-associated ER membranes (MAMs), specialized domains that contribute to the formation and function of MERCSs, where it participates in inter-organelle communication.

The disruption of MERCSs has been implicated in aging, diabetes, and several neurodegenerative disorders, indicating that these structures actively contribute to disease pathogenesis rather than functioning solely as structural interfaces [33]. Accumulating evidence further suggests that CAV-1 influences ER–mitochondrial calcium signaling, lipid trafficking, mitochondrial dynamics, and mitophagy through its association with MAMs/MERCSs, thereby contributing to cellular metabolic homeostasis [33].

However, direct evidence demonstrating that CAV-1-mediated alterations in MERCSs drive diabetes-associated AD pathology remains limited. Most available studies have been performed in cultured cells or non-neuronal experimental systems, and only a few have examined these mechanisms in diabetes-related models of neurodegeneration [33,34]. Consequently, the proposed CAV-1–MERCS–mitophagy axis should currently be regarded only as a promising mechanistic hypothesis rather than an established pathway. Future studies employing cell-type-specific genetic approaches and clinically relevant models will be essential to establish causal relationships. Nevertheless, current evidence supports altered MERCS function as a plausible mechanism linking diabetes-induced metabolic stress to mitochondrial dysfunction and AD-related neurodegeneration [35].

## 6. CAV-1, Mitophagy, and Mitochondrial Quality Control

Mitochondrial dysfunction and impaired mitophagy are increasingly recognized as common pathological features of both T2D and AD. Mitochondria–endoplasmic reticulum contact sites (MERCSs) function as important signaling platforms that regulate mitochondrial dynamics, calcium homeostasis, lipid metabolism, and mitochondrial turnover through interactions with multiple tethering proteins and regulatory pathways.

Emerging evidence suggests that CAV-1 may contribute to mitochondrial quality control by maintaining mitochondrial homeostasis and influencing mitophagy-related pathways. Loss of CAV-1 has been associated with impaired mitochondrial turnover, increased oxidative stress, and enhanced cellular vulnerability to metabolic stress [24]. These findings suggest that defective mitochondrial quality control may represent at least one mechanism through which CAV-1 dysfunction contributes to neurodegenerative processes under diabetic conditions.

However, direct evidence establishing a causal relationship between CAV-1 deficiency, impaired mitophagy, and diabetes-associated AD pathology remains limited. Most available studies have examined mitochondrial responses in cellular or non-neuronal models, and further investigation using neuron- and cell-type-specific approaches will be required to define the contribution of CAV-1 to mitophagy regulation in vivo.

## 7. CAV-1, Neuroinflammation, and Innate Immune Signaling

Chronic low-grade inflammation is a fundamental pathogenic mechanism shared by T2D and AD. Persistent hyperglycemia, insulin resistance, and dyslipidemia promote systemic inflammatory responses that extend to the central nervous system, where they contribute to microglial activation, synaptic dysfunction, and progressive neuronal injury [6,15].

Among inflammatory pathways potentially influenced by CAV-1, nuclear factor kappa B (NF-κB) signaling has received considerable attention. NF-κB is a central transcriptional regulator of inflammatory responses that controls the expression of numerous cytokines, chemokines, and adhesion molecules [36]. Hyperglycemia and cellular stress activate NF-κB, resulting in increased production of pro-inflammatory mediators, including tumor necrosis factor-α (TNF-α), interleukin-1β (IL-1β), and interleukin-6 (IL-6) [37]. Experimental studies suggest that CAV-1 can negatively regulate NF-κB activation, whereas CAV-1 deficiency may enhance inflammatory signaling and increase susceptibility to inflammatory injury [38,39,40].

Recent studies have also implicated the NLRP3 inflammasome as an important mediator linking metabolic dysfunction to neurodegeneration [41]. Hyperglycemia, oxidative stress, and mitochondrial dysfunction promote NLRP3 activation, resulting in maturation and release of IL-1β and IL-18. Excessive inflammasome activation has been documented in both T2D and AD and contributes to synaptic dysfunction, neuronal injury, and cognitive decline.

Emerging evidence suggests that CAV-1 may influence inflammasome-related pathways through the regulation of membrane microdomain organization, oxidative stress responses, and NF-κB signaling [42,43]. Nevertheless, current evidence remains largely indirect, and reliable demonstration that CAV-1 regulates NLRP3 inflammasome assembly or activation in diabetes-associated AD models is currently lacking. Therefore, the proposed CAV-1–NLRP3 pathway should be considered a potential mechanistic link requiring further experimental validation rather than an established signaling mechanism.

Collectively, available evidence supports the possibility that CAV-1 dysfunction contributes to neuroinflammation by altering NF-κB-dependent inflammatory responses, influencing microglial activation, and modifying cytokine-mediated neuronal injury. Further clarification of CAV-1-dependent immune mechanisms may reveal new opportunities for biomarker development and therapeutic intervention in diabetes-associated neurodegeneration [40].

## 8. Therapeutic Targeting of CAV-1

Accumulating experimental evidence suggests that the restoration of neuronal CAV-1 expression can attenuate cognitive impairment, preserve synaptic integrity, and partially reverse disease-associated transcriptomic alterations in experimental models of AD [19]. Because of its involvement in insulin signaling, mitochondrial homeostasis, neurovascular regulation, and inflammatory responses, CAV-1 has emerged as a potential therapeutic target at the intersection of T2D and AD [10,31].

Several therapeutic strategies targeting CAV-1-associated pathways are currently being explored. Gene therapy approaches using viral vectors to increase neuronal CAV-1 expression have demonstrated encouraging preclinical effects, including improved synaptic function and reduced neurodegenerative changes [10,24]. These studies represent the strongest current evidence supporting direct therapeutic targeting of CAV-1. On the other hand, pharmacological approaches aimed at modifying caveolae organization or membrane signaling may influence CAV-1-dependent pathways by restoring receptor localization and improving insulin signaling, although their effects on CAV-1 itself remain incompletely defined.

Strategies targeting mitochondrial dysfunction and MERCS abnormalities may provide additional therapeutic benefit by improving calcium homeostasis, mitochondrial bioenergetics, and autophagic function [44]. Similarly, mitochondrial protective compounds, antioxidants, and agents that regulate mitochondrial dynamics may reduce cellular consequences associated with CAV-1 dysfunction, although these approaches should be considered indirect modulators of CAV-1-related pathways rather than CAV-1-specific therapies [45].

Metabolic interventions, including insulin-sensitizing agents such as metformin and glucagon-like peptide-1 receptor agonists, have demonstrated neuroprotective effects in experimental and clinical studies. However, whether these benefits are mediated through restoration of CAV-1 signaling remains unclear. Likewise, therapies targeting chronic neuroinflammation and abnormal microglial activation may interrupt pathogenic pathways associated with both metabolic dysfunction and neurodegeneration but cannot currently be classified as CAV-1-directed interventions [46].

## 9. Hierarchy of Therapeutic Evidence

Current therapeutic evidence supporting CAV-1 as a target remains predominantly preclinical in nature. The strongest evidence derives from studies directly restoring neuronal CAV-1 expression, which demonstrate improved neurotrophin receptor signaling, preservation of dendritic architecture, enhanced synaptic plasticity, and improved cognitive performance in AD mouse models. In comparison, interventions involving insulin sensitizers, mitochondrial protective agents, anti-inflammatory therapies, membrane microdomain modulation, or MERCS-targeted approaches should be considered only as indirect regulators of pathways in which CAV-1 participates. Although these approaches may converge mechanistically with CAV-1-dependent signaling, direct evidence demonstrating that their therapeutic effects are mediated through CAV-1 remains limited.

Similarly, although altered CAV-1 expression has been associated with AD pathology, the utility of CAV-1 as a clinical biomarker has not yet been established. Prospective human studies are required to determine whether CAV-1 levels in brain tissue, cerebrospinal fluid, plasma, or extracellular vesicles can reliably predict disease progression or therapeutic response.

## 10. Biomarker Potential of CAV-1

Emerging evidence suggests that CAV-1 may serve as a clinically useful biomarker for diabetes-associated cognitive impairment and AD progression. Altered CAV-1 levels in plasma, cerebrospinal fluid, and extracellular vesicles have been associated with abnormalities in membrane signaling, neurovascular integrity, and inflammatory activation, all of which represent early events in metabolic and neurodegenerative disorders [10,27].

Particular attention has focused on extracellular vesicle-associated CAV-1 as a minimally invasive indicator of neuronal and endothelial dysfunction. Caveolin-containing extracellular vesicles participate in intercellular communication between metabolic, vascular, and neural tissues and may reflect systemic disease processes before the onset of overt clinical manifestations [46,47]. Because CAV-1 regulates blood–brain barrier integrity, insulin transport, and inflammatory signaling, changes in circulating CAV-1 levels may provide insight into the progression of diabetes-associated neurovascular dysfunction.

Rather than functioning as a stand-alone biomarker, CAV-1 may prove most valuable when incorporated into multimodal biomarker panels together with established AD biomarkers, including the Aβ42/40 ratio, phosphorylated tau, neurofilament light chain, and markers of endothelial injury [48,49,50]. However, prospective longitudinal studies and standardized analytical methods will be required before CAV-1 can be implemented in clinical practice.

**Future perspectives on CAV-1 as a biomarker.** Although numerous experimental studies implicate CAV-1 in insulin signaling, membrane organization, synaptic function, and neurodegenerative mechanisms relevant to both T2D and AD [15,21], current evidence is insufficient to support CAV-1 as a clinically validated biomarker. To date, there is no robust clinical evidence demonstrating that plasma-, CSF-, or extracellular vesicle-associated CAV-1 reliably identifies diabetic cognitive impairment, predicts Alzheimer’s disease progression, or monitors therapeutic response in patients with T2D-associated AD. Consequently, CAV-1 should presently be regarded as a promising candidate biomarker whose clinical utility remains to be established. Future longitudinal studies integrating standardized biofluid measurements with cognitive assessment, neuroimaging, and established AD biomarkers will be necessary to determine its diagnostic, prognostic, and predictive value.

## 11. Future Directions

Despite considerable progress, several important questions remain unresolved. The cell-specific functions of CAV-1 in neurons, astrocytes, microglia, and endothelial cells require further investigation, as do the mechanisms regulating its activity within lipid rafts and mitochondria–endoplasmic reticulum contact sites. Likewise, the interactions between CAV-1 and major AD susceptibility pathways—including APOE4, tau pathology, and sex-dependent biological differences—remain incompletely understood.

Future studies should also determine how CAV-1 integrates metabolic stress, mitochondrial dysfunction, neurovascular impairment, and innate immune activation throughout the different stages of disease progression. Addressing these questions will require cell-type-specific genetic models, longitudinal human studies, and multi-omics approaches capable of capturing dynamic molecular changes. Such studies will be essential for translating experimental findings into clinically effective diagnostic and therapeutic strategies.

## 12. Limitations of the Current Evidence

Several limitations should be considered when interpreting the current evidence. Most mechanistic insights have been obtained from in vitro studies and genetically modified animal models, which do not fully reproduce the complexity of human diabetes or AD. Furthermore, global CAV-1 deficiency affects multiple organs and cell types, making it difficult to distinguish direct tissue-specific effects from secondary systemic consequences. Variability in experimental design, disease stage, analytical methods, and outcome measures further contributes to inconsistent findings across studies.

Accordingly, future research should emphasize standardized experimental protocols, cell-type-specific models, and well-powered longitudinal clinical studies. These efforts will be necessary to clarify the context-dependent functions of CAV-1 and to determine its potential as a candidate biomarker and a potential therapeutic target.

## 13. Conclusions

CAV-1 has emerged as an important regulator of membrane organization and signal transduction at the intersection of T2D and AD. The evidence reviewed in this article indicates that CAV-1 influences multiple cellular processes implicated in both disorders, including insulin signaling, lipid raft organization, mitochondrial function, oxidative stress, neuroinflammation, and synaptic plasticity. These findings support the concept that CAV-1 may represent a mechanistic link between metabolic dysfunction and neurodegeneration.

However, the strength of the available evidence varies considerably. Most data supporting a role for CAV-1 are derived from in vitro studies, animal models, and mechanistic investigations, whereas direct evidence from well-characterized human clinical studies remains limited. Likewise, although neuronal CAV-1 restoration has demonstrated neuroprotective effects in preclinical models, these findings should not yet be interpreted as evidence for an established therapeutic strategy in patients. Similarly, while altered CAV-1 expression has been associated with AD pathology, its potential as a biomarker for diabetic cognitive impairment, AD progression, or therapeutic response has not been clinically validated.

Overall, current evidence supports CAV-1 as a promising mechanistic target for further investigation rather than as an established therapeutic or diagnostic tool. Future studies should focus on defining the cell type-specific and disease stage-dependent regulation of CAV-1, elucidating its interactions with insulin signaling and mitochondrial homeostasis, and validating its clinical relevance in longitudinal studies involving patients with T2D, AD, and T2D-associated AD. Such investigations will be essential for determining whether the modulation of CAV-1 can be translated into effective diagnostic or therapeutic strategies.

This review summarizes current evidence supporting a central role for caveolin-1 (CAV-1) in the molecular interplay between type 2 diabetes and Alzheimer’s disease. Experimental and clinical studies collectively indicate that CAV-1 regulates multiple biological processes implicated in both disorders, including insulin signaling, lipid metabolism, neurovascular function, mitochondrial homeostasis, autophagy, and innate immune responses. Rather than acting through a single mechanism, CAV-1 appears to coordinate an interconnected network of signaling pathways that links systemic metabolic dysfunction with neurodegeneration.

At the same time, important inconsistencies remain across the literature. Reported alterations in CAV-1 expression and function vary according to cell type, brain region, disease stage, and experimental model, indicating that CAV-1 functions as a context-dependent regulator rather than a uniformly protective or detrimental molecule. Resolving these discrepancies will require standardized experimental approaches and carefully designed translational studies.

Current mechanistic knowledge is derived predominantly from cellular and animal models, whereas evidence from human studies remains comparatively limited. Consequently, although CAV-1 represents a promising therapeutic target and biomarker candidate, its clinical relevance has not yet been fully established. Future investigations should prioritize validation in well-characterized patient cohorts, integration of multi-omics datasets, and development of biomarkers capable of identifying individuals at increased risk of diabetes-associated cognitive decline.

## Data Availability

No new data were generated or analyzed in this study. Data supporting this review are available from the cited references.
